# Differences in Depression and Anxiety, Quality of Life and Quality of Sleep Between Neuropathic and Nociceptive Patients with Chronic Low Back Pain: A Comparative, Multi-Center, Cross-Sectional Study

**DOI:** 10.3390/healthcare14131905

**Published:** 2026-07-01

**Authors:** Ivan Beljan, Ivan Krakan, Josip Šimić, Hrvoje Ajman, Zoran Špoljarić

**Affiliations:** 1 Faculty of Health Studies, University of Mostar, 88000 Mostar, Bosnia and Herzegovina; 2 Faculty of Kinesiology, University of Zagreb, 10000 Zagreb, Croatia; 3 Faculty of Kinesiology, Josip Juraj Strossmayer University of Osijek, 31000 Osijek, Croatia

**Keywords:** chronic lumbar pain, neuropathic pain, nociceptive pain, depression, anxiety, quality of life

## Abstract

**Background:** The available evidence highlights that low back pain (LBP) can be categorized into neuropathic or nociceptive pain, according to mechanical- or lesion/disease-related issues. Both types of pain are associated with increased psychological and sleep problems and well-being. However, little is known about how a specific type of LBP pain (neuropathic vs. nociceptive) has an impact on these lifestyle factors. Therefore, the main purpose of the study was to examine the differences in depression and anxiety symptoms, quality of life (QoL) and quality of sleep in patients with LBP. **Materials and Methods:** In this cross-sectional study, we included 150 patients [mean ± standard deviation (SD) age: 46.54 ± 13.36 years; height: 178.06 ± 10.80 cm; weight: 85.26 ± 15.53 kg; body mass index (BMI): 26.77 ± 3.63 kg/m^2^; 45.0% women] from three centers in Bosnia and Herzegovina and Croatia between July and October 2025. All patients suffered from chronic LBP confirmed by computerized tomography (CT). The classification of pain was conducted using the Leeds Assessment of Neuropathic Symptoms and Signs (LANNS), the Douleur Neuropathique 4 (DN4) and the PainDETECT (PD-Q) questionnaires. Positive scores in all three questionnaires denoted neuropathic pain. Factors of depression (Beck’s Depression Inventory), anxiety [Hamilton Anxiety Rating Scale (HARS)], QoL (SF-36 scale) and sleep quality [Pittsburgh Sleep Quality Index (PSQI)] were also collected. **Results:** After adjusting for sociodemographic covariates, neuropathic patients reported higher depression [*F*_1,149_ = 7.790, *p* = 0.006, partial eta squared (ηp^2^) = 0.051] and anxiety level scores (*F*_1,149_ = 8.140, *p* = 0.005, ηp^2^ = 0.053), lower QoL (*F*_1,149_ = 19.088, *p* < 0.001, ηp^2^ = 0.116) and poorer sleep quality (*F*_1,149_ = 19.654, *p* < 0.001, ηp^2^ = 0.119). **Conclusions:** In summary, this study shows that neuropathic patients with LBP are more depressed and anxious, and have lower levels of QoL and poorer sleep quality, in comparison to nociceptive patients. The findings suggest that these lifestyle factors need to be considered when establishing the appropriate rehabilitation and management of LBP.

## 1. Introduction

Low back pain (LBP) encompasses a group of heterogeneous diseases and disorders that the World Health Organization defines as pain located “below the lowest costal margin and above the inferior gluteal folds, with or without pain radiating into the leg” [1]. It represents a major global public health problem and one of the leading causes of disability worldwide, affecting more than 600 million people worldwide by [2,3], and it is predicted that the incidence of low back pain will increase by 5% by 2050, reaching over 800 million people [2]. In the United States, the prevalence of LBP pain is estimated at 13.1%, increasing with age and reaching 27.4% among individuals aged 50–59 years, while the prevalence in Europe reaches around 40% [4]. Findings suggest that being a female of the White race, irrespective of sex, low socioeconomic status and being married increase the odds for LBP [1,5]. Another negative impact of LBP targets economic costs, ranging from 2.0 to 5.0 billion U.S. dollars annually for its management and rehabilitation in developing and developed countries [5,6,7].

Low back pain is considered a complex clinical syndrome that may involve different pain mechanisms, including neuropathic pain, nociceptive pain, or a combination of both mechanisms, commonly referred to as mixed pain [8,9]. Neuropathic pain in LBP is defined as pain caused by a lesion or disease affecting the somatosensory nervous system and may occur with or without a nociceptive component [10]. In contrast, evidence defines nociceptive pain as a ‘result of activity in neural pathways caused by tissue injury or potential tissue-damaging stimuli such as surgery and arthritis’ [11]. Both neuropathic and nociceptive mechanisms contribute to the clinical presentation of LBP and associated leg pain, where leg pain is often attributed to neuropathic mechanisms, while axial back pain is more frequently associated with nociceptive processes [12]. In clinical practice, approximately 20% of patients with LBP experience neuropathic pain [10].

LBP that persists for >3 months, is considered chronic [13]. Along with an increased number of physical symptoms, chronic LBP has been constantly associated with psychological factors of depression and anxiety [14,15,16], and behavioral-related outcomes, including quality of life (QoL) [17,18,19] and sleep [20,21,22]. In particular, both cross—sectional and longitudinal data have confirmed the hypothesis that patients with chronic LBP experience higher levels of depression and anxiety, and reduced qualities of life and sleep [14,15,16,17,18,19,20,21,22]. Although previous evidence on the aforementioned topic already exists, fewer studies have examined differences in psychological and behavioral correlates between neuropathic vs. nociceptive chronic LBP in adults [11,23,24,25,26,27,28]. A study by Hiyama et al. [11] showed that patients with neuropathic chronic LBP exhibited poorer scores in mental health, irrespective of the presence of leg pain. Similar observations were noted in another two studies, where neuropathic patients reported lower QoL and mental functioning [27,28]. On the other hand, results by de Souza et al. [29] indicated that the type of pain (neuropathic vs. nociceptive) had no role in depression, anxiety and sleep disorders, indicating that when compared to healthy participants, patients with neuropathic and nociceptive pain would have similar psychological outcome levels.

Based on previous evidence, it can be concluded that findings are still inconclusive, due to the fact that only a handful of studies categorized patients according to the type of pain [11,27,28,29]. Nevertheless, available data suggests that more psychological and behavioral disorders affect patients with neuropathic chronic LBP [11,27,28]. As highlighted from a physiological and biochemical perspective, neuropathic pain arises from injury or dysfunction on a neural basis [12], often accompanied by structural changes of neural ends [30]. On the other hand, nociceptive pain ‘originates from the activation of peripheral nociceptors in response to noxious mechanical, thermal, or chemical stimulus’ [12]. However, in comparison to neuropathic pain, nociceptive patients often report a localized pain associated with tissue injury or inflammation and proportional to the stimuli [12], which makes diagnosis and treatment easier to establish and implement during the rehabilitation process. This would suggest that nociceptive pain associated with locomotor discomfort and dysfunction may be more restorable and less challenging to detect and treat, which has direct implications for better psychological and behavioral functioning [12]. Despite this information, there is no clear consensus as to whether patients with neuropathic chronic LBP may be more vulnerable to depression and anxiety symptoms, and lower levels of QoL and sleep. Second, the classification of pain as neuropathic and nociceptive has yet to be investigated, since previous studies have used a variety of self-reported questionnaires to distinguish between them [11,12,24]. The results of this study may give a new insight into which modifiable factors differ between neuropathic and nociceptive chronic LBP, and how these differences can be practically applied in healthcare systems. If findings support the premise that neuropathic patients have higher odds for depression and anxiety, and reduced levels of QoL and sleep, practitioners may consider using psychological and behavioral components of health for pain treatment and management.

Therefore, the main purpose of the present study was to examine the differences in depression and anxiety levels, QoL and sleep outcomes between neuropathic and nociceptive patients with chronic LBP. Based on previous findings [11,12], we hypothesized that a higher prevalence of patients with chronic LBP would be classified as having nociceptive pain, and those classified as neuropathic patients would exhibit higher levels of depression and anxiety, and poorer QoL and sleep domains.

## 2. Materials and Methods

### 2.1. Participants

In this observational, cross-sectional study, we included 150 patients with chronic LBP [mean ± standard deviation (SD) age: 46.54 ± 13.36 years; height: 178.06 ± 10.80 cm; weight: 85.26 ± 15.53 kg; body mass index (BMI): 26.77 ± 3.63 kg/m^2^; 45.0% women] recruited from the following institutions: Physical Therapy and Rehabilitation Beljan in Tomislavgrad, Physiotherapy Sušac in Čapljina and the Health Center in Metković—Department of Physical Medicine and Rehabilitation. The study included patients of both sexes aged 18–65 years. Classification of pain was done by using the Leeds Assessment of Neuropathic Symptoms and Signs (LANNS) [31,32,33,34], the Douleur Neuropathique 4 (DN4) [35,36,37,38] and the PainDETECT (PD-Q) questionnaires [39,40,41]. Positive scores in all three questionnaires denoted neuropathic pain. Based on a cross-tabulation matrix, patients were categorized into neuropathic (n = 62) and nociceptive pain (n = 88). The utility of using different questionnaires for assessing different types of pain was explained in a later text. Based on the G*power calculator and *a priori* analysis to determine the required sample size and using medium effect size (ES = 0.34) obtained in previous studies between neuropathic and nociceptive patients in mental health, *p* < 0.05, statistical power of 1 − β = 0.95, two groups (n = 2) and three covariates (please see Section 2.4), the appropriate total sample size was n = 138. The study was conducted from July to October 2025, for a period of 3 months. The study was approved by the Ethics Committee of the Metković Primary Health Care Center (Croatia) on 23 September 2024 (approval no: 02-919). The study was conducted in accordance with the principles of the Declaration of Helsinki. All participants provided written informed consent prior to participation in the study.

### 2.2. Procedures

All patients were verbally informed about the purpose and hypothesis of the study and then signed informed consent to participate in the study. The study was conducted by fellow physiotherapists and physicians with >10 years of experience in clinical practice. Exclusion criteria for patient recruitment were: (i) patients with LBP for less than 3 months, (ii) chronic LBP in combination with other musculoskeletal diseases, (iii) systemic medical disease such as heart disease, chronic renal failure, fibromyalgia, and dysthyreosis, spinal surgery, primary psychiatric disorder such as depression, anxiety, or insomnia, and (iv) a person who was unable to understand and answer the questionnaires. To omit a potential bias and secure anonymity, questionnaires were randomly given to all patients without their identification information, but with a unique code.

### 2.3. Instruments

**Sociodemographic parameters**: To examine these factors, we used sex (men vs. women), age (years), height (cm), weight (kg), body mass index (BMI; kg/m^2^), marital status (single, married, divorced and widowed), the level of education (primary education, secondary education, faculty—3 and 5 years, and a doctorate), current occupation (employed, unemployed, householder, pensioner), and socioeconomic status (below average, average and above average). Height and weight were measured using objective and standardized procedures of a stadiometer (Seca, Germany) and a digital scale with a precision of 0.1 cm and 0.1 kg. BMI was calculated with the following formula: [weight (kg)/height (cm)^2^]. We classified BMI according to the International Obesity Task Force (IOTF) [42] into underweight (<18.5 kg/m^2^), normal weight (18.5–24.9 kg/m^2^), overweight (25.0–29.9 kg/m^2^) and obesity (≥30.0 kg/m^2^).

**Visual analog scale (VAS)**: To examine the validity of the LANNS, the DN-4, and the PDQ, we correlated total scores of all three questionnaires with the present VAS, a 10-cm pain rating scale that represented ‘a continuum between the two ends of the scale—“no pain” on the left end (0 cm) of the scale and the “worst pain” on the right end of the scale (10 cm)’ [43]. The final score was recorded in centimeters starting from the left end of the scale and indicated the pain severity.

The assessment of the type of pain was measured using the three questionnaires: the LANNS, the DN-4, and the PDQ [31,32,33,34,35,36,37,38,39,40,41]. We previously highlighted that positive scores in all questionnaires denoted neuropathic pain. The reason for such an approach was the fact of previously reported findings, where combining those three questionnaires might strengthen the detection of neuropathic pain in people with acute whiplash-associated disorders [44]. A study by Ríos-León et al. [44] concluded that these questionnaires should be considered when screening for neuropathic pain in the aforementioned patients. When the scores of all three questionnaires were combined, the correlation with the VAS was stronger (*r* = 0.58, *p* < 0.001), compared to separate questionnaire assessments, which were presented in a further text. Cross-tabulation data between the questionnaires is presented in Section 3.

**LANSS pain scale**: The questionnaire was based on sensory description analysis and sensory dysfunction assessment and provides immediate information in clinical settings. The LANSS was developed to differentiate pain of predominantly neuropathic or nociceptive origin. The scale consisted of seven items, grouped sensory description and sensory examination with a simple scoring system, and a score of ≥12 on the LANSS scale indicated the presence of neuropathic pain [31,32,33,34]. The reliability of the questionnaire (Cronbach’s α = 0.88) and validity against the VAS (*r* = 0.39, *p* < 0.001) were satisfactory.

**DN-4 questionnaire**: This questionnaire contained seven items related to symptoms (e.g., burning, electric shock, painful cold, tingling, prickling, numbness, itching) and three items related to physical examination (hypoesthesia to brushing, assessed with a Somedic brush, hypoesthesia to fine tactile stimuli, with each item scored as present 1 point or absent 0 points). It was used to measure the prevalence of neuropathic pain in the general population and specific clinical conditions (such as diabetic neuropathy), and a score greater than 4 out of 10 points indicated the presence of neuropathic pain [35,36,37,38]. Similar to the LANNS, the DN-4 showed satisfactory reliability (Cronbach’s α = 0.83) and validity (*r* = 0.48, *p* < 0.001) properties.

**PDQ:** A symptom-based assessment tool developed to aid in the identification of neuropathic pain [39,40,41]. It assigned patients a score that classified their pain into three groups: neuropathic, vague, or nociceptive pain. The total PDQ score was obtained by summing the scores of the seven sensory items, the radiation score, and the pain pattern score. The total score ranged from −1 to 38 points. A score of ≤18 points indicated nociceptive pain, and a score of ≥19 points indicated a high probability of neuropathic pain [39,40,41]. The questionnaire was a reliable (Cronbach’s α = 0.86) and valid (*r* = 0.52, *p* < 0.001) tool to assess neuropathic pain.

To assess the level of depression and anxiety, we used Beck’s Depression Inventory (BDI) [45,46] and the Hamilton Anxiety Rating Scale (HAMS) [47,48].

**BDI:** The questionnaire was a 21-item self-report tool to assess the severity of depressive symptoms, based on the Diagnostic and Statistical Manual of Mental Disorders (DSM-IV). The questionnaire consisted of 21 items with multiple response options displayed on a scale from 0 to 3, depending on the severity of symptoms within the two weeks prior to administration of the questionnaire. The minimum and maximum scores ranged from 0 to 63, with higher scores indicating greater symptom severity. Scores of 0–13 indicated minimal severity, 14–19 mild severity, 20–28 moderate severity, and 29–63 severe depression [45,46]. The reliability of the questionnaire in this study was α = 0.88.

**HAMS**: It consisted of 14-symptom-defined items and addressed both psychological and somatic symptoms, including anxious mood, tension, fears, insomnia, depressed mood, somatic symptoms, cardiovascular, respiratory, gastrointestinal, genitourinary, autonomic, and observed interview behavior. Each item was scored on a numerical scale from 0 (not present) to 4 (severe): >0–7 no or minimal anxiety, 8–14 mild anxiety, 15–23 moderate anxiety, and ≥24 severe anxiety [47,48]. Cronbach’s α showed excellent reliability properties (0.90).

QoL was measured by the 36-Item Short Form Health Survey (SF-36), a reliable and valid tool to examine different components of QoL [49,50].

**SF-36 scale**: The scale consisted of 36 items covering eight subscales related to physical and social functioning, physical and emotional difficulties, vitality (energy), mental and general health, and pain. The score on each subscale ranged from 0 (worst possible state) to 100 (best possible state), with higher scores indicating better health-related QoL. It is a tool widely recognized for its robustness and validation across populations and diseases, and it has demonstrated good psychometric properties, with high internal consistency and the ability to detect changes in perceived health over time [49,50]. The reliability of the questionnaire in this study was α = 0.85.

Finally, the assessment of sleep quality was monitored using the Pittsburgh Sleep Quality Questionnaire (PSQI) [51,52].

**PSQI**: Assessed as one of the most commonly used questionnaires for measuring sleep quality and sleep disturbances over a period of more than 1 month [51,52]. The questionnaire had seven subscale scores out of nineteen individual items. Each subscale was scored from 0 to 3. The total PSQI score ranged from 0 to 21, with higher scores indicating poorer sleep quality. A total PSQI score greater than 5 has been shown to be highly sensitive and specific in distinguishing good sleepers from poor sleepers in a number of populations [51,52]. The questionnaire presented satisfactory reliability properties in this study (Cronbach’s α = 0.79).

### 2.4. Statistical Analysis

To test data normality, a Kolmogorov–Smirnov (K-S) test was used. Data which followed normal distribution were presented as arithmetic mean (AS) and standard deviation (SD) and those with skewed curves as median. Baseline differences in sociodemographic characteristics between nociceptive and neuropathic patients were determined with Student’s *t*-test for independent samples or Mann–Whitney U-test. If we observed significant differences in sociodemographic data, the existing variables were further included in models as covariates. Of note, we obtained significant differences in sex ratio, BMI and educational level between patients with different types of pain. The univariate analysis of covariance (ANCOVA) was used to calculate differences between nociceptive and neuropathic patients in depression and anxiety levels, QoL and quality of sleep. A measure of effect size (ES) was presented as partial eta squared (ηp^2^) with the following cut-off points: (i) 0.01—small ES, (ii) 0.06—medium ES, and (iii) 0.14—large ES [53]. Categorical data were calculated by using 2 × 2 contingency tables with a Chi-square test. For the purpose of this study, Hedge’s *g* and Kraemer’s *V* tests were used to examine ESs in numerical and categorical data. All procedures performed in this study were calculated using the Statistical Packages for Social Sciences ver. 28 (SPSS, IBM Statistics, Chicago, Il, USA). Significance was set *a priori* to *p* < 0.05, and it was two-sided.

## 3. Results

Before further analysis, we examined center-related differences in socioeconomic characteristics, pain outcomes from the LANNS, DN-4 and PDQ questionnaires, and VAS. Also, we observed differences in depression and anxiety, QoL and quality of sleep. The results of ANOVA showed no significant differences between the patients recruited from the three centers (*p* > 0.05).

Second, we calculated cross-tabulation matrices between the LANNS, DN-4 and PDQ questionnaires. When using the LANNS, 55 patients (36.7%) were classified as having neuropathic pain, 67 patients (44.7%) were neuropathic when using the DN-4, and 78 patients (52.0%) were neuropathic when PDQ was administered. When comparing the LANNS and DN-4 questionnaires, 74.6% of patients were correctly classified as neuropathic (sensitivity), while 94.0% of patients were nociceptive (specificity) (kappa = 0.71, *p* < 0.001). Lower sensitivity (neuropathic pain detection) was determined between the LANNS and PDQ (61.5%), while similar specificity (nociceptive patients) of 90.3% was shown (kappa = 0.54, *p* < 0.001). Finally, sensitivity between the DN4 and PDQ was 74.4% and specificity of 87.5% (kappa = 0.62). When all three questionnaires were combined and patients were categorized as neuropathic vs. nociceptive, 62 patients (41.3%) had neuropathic pain.

Basic descriptive statistics of the total sample and according to the pain-related outcome (nociceptive vs. neuropathic) are presented in Table 1. A larger proportion of men suffered from neuropathic type of pain, compared to women (*p* = 0.002). Also, neuropathic patients were heavier and had higher BMI, compared to nociceptive patients (*p* < 0.05). More neuropathic patients had a significantly higher proportion of overweight and obese individuals, and a lower percentage of normal-weight individuals, in comparison to nociceptive patients. Also, nociceptive participants were more highly educated; >50.0% of them had completed higher education in any form (*p* = 0.048). Other sociodemographic variables were not statistically significant between the pain types.

Differences in anxiety and depression between the study participants with nociceptive and neuropathic pain are presented in Table 2. After adjusting for age, BMI and educational level, neuropathic patients reported higher depression (*F*_1,149_ = 7.790, *p* = 0.006, ηp^2^ = 0.051) and anxiety level scores (*F*_1,149_ = 8.140, *p* = 0.005, ηp^2^ = 0.053) when using numerical data. When the BDI and HARS were classified into severity of depression and anxiety, a higher percentage of neuropathic participants suffered from moderate and severe depression and moderate to severe and severe to very severe anxiety, compared to the nociceptive participants.

Differences in the SF-36 domains between nociceptive and neuropathic patients are shown in Table 3. Neuropathic patients yielded poorer physical functioning (*F*_1,149_ = 5.267, *p* = 0.023, ηp^2^ = 0.035), more difficulties in both physical (*F*_1,149_ = 9.567, *p* = 0.002, ηp^2^ = 0.062) and emotional roles (*F*_1,149_ = 9.027, *p* = 0.003, ηp^2^ = 0.059), less vitality (energy) (*F*_1,149_ = 4.769, *p* = 0.030, ηp^2^ = 0.031), poorer mental health (*F*_1,149_ = 3.512, *p* = 0.043, ηp^2^ = 0.024), more bodily pain (*F*_1,149_ = 12.986, *p* < 0.001, ηp^2^ = 0.082) and lower overall SF-36 score (*F*_1,149_ = 18.149, *p* < 0.001, ηp^2^ = 0.111), opposed to nociceptive patients. No significant differences in social functioning (*F*_1,149_ = 1.449, *p* = 0.231, ηp^2^ = 0.010) and general health (*F*_1,149_ = 1.247, *p* = 0.266, ηp^2^ = 0.009) between the two groups were observed.

Table 4 shows differences in PSQI domains between nociceptive and neuropathic patients. Neuropathic patients exhibited a shorter sleep duration (*F*_1,149_ = 6.843, *p* = 0.010, ηp^2^ = 0.045), less sleep efficiency (*F*_1,149_ = 7.829, *p* = 0.006, ηp^2^ = 0.051), more sleep disturbances (*F*_1,149_ = 13.506, *p* < 0.001, ηp^2^ = 0.085), the use of more sleep medication (*F*_1,149_ = 8.969, *p* = 0.003, ηp^2^ = 0.058), poorer sleep quality (*F*_1,149_ = 16.595, *p* < 0.001, ηp^2^ = 0.103) and higher sleep dysfunction (*F*_1,149_ = 19.466, *p* < 0.001, ηp^2^ = 0.118), compared to nociceptive patients. The global PSQI score indicated large differences between the nociceptive and neuropathic patients, where neuropathic patients showed significantly poorer sleep quality when observed as numerical (*F*_1,149_ = 17.021, *p* < 0.001, ηp^2^ = 0.105) or categorical outcomes (*F*_1,149_ = 30.196, *p* < 0.001, ηp^2^ = 0. 172).

## 4. Discussion

The main purpose of this study was to examine the differences in some psychological (depression and anxiety) and behavioral outcomes (QoL and sleep quality) in neuropathic and nociceptive patients with chronic LBP using three validated questionnaires (LANSS, DN4, and PDQ). The findings suggest that neuropathic patients exhibit higher levels of depression and anxiety and lower QoL and sleep quality. The largest magnitudes of these differences are observed for overall SF-36 and quality of life scores, followed by sleep dysfunction, more bodily pain and sleep disturbances.

To the best of our knowledge, no previous study has simultaneously used these three questionnaires to assess neuropathic pain in patients with chronic LBP. Since we confirmed that the combination of the LANNS, DN-4 and PDQ to detect patients with neuropathic pain was more strongly correlated to the VAS, in comparison to separate correlations, we speculated that using these tools in clinical practice would yield better discriminative ability between the types of pain. Unfortunately, the consensus of the most appropriate questionnaire to determine neuropathic pain has yet to be investigated. Another reason for including the data from all three questionnaires was that there was only moderate agreement between them, where the rest of the variance should be attributed to other factors. The results of this study indicated that neuropathic patients had higher levels of depression and anxiety, opposed to nociceptive patients, which is in line with previous studies [11,12,27,28,54,55]. In a sample of 239 patients with LBP, Hiyama et al. [11] compared neuropathic and nociceptive patients in the QoL using the SF-36 scale and found that neuropathic patients had lower mental health scores (38.5), while nociceptive patients exhibited higher values (48.0). Similar observations were noted between neuropathic and nociceptive patients with and without leg pain, where mental health status remained poorer in the neuropathic patients [11]. A study by Ohtori et al. [27] using the Japanese Orthopaedic Association Back Pain Evaluation Questionnaire (JOABPEQ) showed that mental health was worse in patients with neural disc impairments, while patients with disc degeneration exhibited higher mental health values. Similarly, a study by Beith et al. [28] categorized patients with LBP into neuropathic and nociceptive according to the PDQ and found that patients with neuropathic pain components had significantly higher depression and anxiety levels (assessed by the HARS questionnaire), and poorer mental health (assessed by the SF-36 scale) than those in the nociceptive group. Other two studies reported that neuropathic patients had more severe co-morbidities, such as depression and anxiety levels, which directly affected functionality and use of healthcare resources [54,55]. In particular, a study by Freynhagen et al. [54] showed that neuropathic patients were 4.5 times more likely to report depression and anxiety disorders than in the nociceptive patients. Thus, based on the results of the aforementioned and previous studies, it could be concluded that neuropathic patients tend to have higher depression and anxiety rates and reduced overall mental health.

We also found that neuropathic patients performed more poorly in the QoL assessed by the SF-36 scale, which is also in line with the previous literature [11,17,18,19,27,28]. Hiyama et al. [11] reported that, in patients with neuropathic pain, all eight scales in the SF-36 showed lower values, regardless of the presence of lower limb symptoms, compared with those for nociceptive pain. Ohtori et al. [27] showed similar findings, where low back pain in patients with disc herniation was significantly worse than that in patients with disc degeneration/spondylosis, indicating more bodily pain. Also, walking ability for patients with neuropathic pain was significantly worse than that for those with nociceptive pain [27]. Finally, social functioning was worse in neuropathic patients than in nociceptive patients [27]. A recent study by Ge et al. [19] indicated that individuals with neuropathic chronic LBP reported poorer physical function, more limitations in performing major life tasks and social activities, more depressive symptoms, and lower health-related quality of life, compared to nociceptive patients. In another study, scores for physical and mental components of the QoL were significantly lower in neuropathic pain patients with both acute/subacute and chronic LBP [54]. Beith et al. [28] also demonstrated that patients with neuropathic pain had lower QoL compared with other pain groups (mixed and nociceptive), with significantly greater pain, disability, anxiety and depression scores. Similar to the differences in depression and anxiety towards neuropathic patients, previous studies have documented that neuropathic patients tend to have lower levels of QoL, especially in the components of bodily pain, physical functioning and overall QoL score.

Finally, our results showed that neuropathic patients had poorer sleep quality when assessed by the PSQ. These findings are relatable to previous evidence, in which neuropathic patients with chronic LBP exhibited more sleep problems and disorders than those with nociceptive pain [20,21,22,54]. A study by Freynhagen et al. [54] determined that a strong inverse correlation between the Medical Outcomes Study sleep scale (MOS) questionnaire and the PDQ, where a gradually higher score in the PDQ led to poorer optimal sleep, more sleep disturbances, a higher level of somnolence, poorer sleep quality and less sleep adequacy. In other words, patients classified as having neuropathic pain reported more impaired and fragmented overall sleep quality [54]. In general, previous systematic reviews have shown that chronic LBP is associated with greater sleep disturbance, reduced sleep duration and sleep quality, increased time taken to fall asleep, poor daytime function, and greater sleep dissatisfaction and distress [20]. However, less evidence has been presented for examining the differences between neuropathic and nociceptive pain.

LBP has been documented as one of the most prevalent musculoskeletal conditions worldwide [1,2,3]. Although the term is well-recognized in everyday literature and clinical practice, evidence often highlights the importance of recognizing different pain mechanisms, including nociceptive and neuropathic, for different and personalized treatment approaches [12,56]. Although LBP is highly prevalent, its pathophysiology is often not well understood. Clinical management of LBP often depends on a structural–anatomical paradigm, which may not effectively address the underlying pain mechanisms [12]. Despite advances in understanding these mechanisms, clinical practice often lacks systematic tools to classify pain in patients with LBP. This lack of systematic tools can result in a failure to identify the dominant pain mechanism, which in turn can lead to inappropriate or ineffective treatment strategies, especially in cases of chronic LBP [12] or a higher prevalence of LBP according to sex, which was confirmed in this study. For example, baseline differences between neuropathic and nociceptive patients revealed significantly larger proportions of neuropathic LBP in men compared to women. However, such findings are not in line with previous data comparing men and women for neuropathic and nociceptive pain [57]. From a biomolecular perspective, ‘microglial activation in the spinal cord plays a dominant role in maintaining hypersensitivity in men, by releasing proinflammatory cytokines and neuromodulators that amplify nociceptive transmission and promote chronic pain’ [57]. Therefore, the neuropathic type of pain is highly related to distinct immune pathways, while nociceptive pain is more hormone-based, where ‘fluctuations in ovarian hormones like estrogen can sensitize excitatory neurotransmission and reduce the efficacy of descending inhibitory circuits, thereby amplifying the central nervous system’s response to otherwise non-noxious stimuli’ [57]. The usage of three reliable and valid questionnaires to detect neuropathic and nociceptive pain represents the strength of this study. However, requiring triple positivity (LANSS, DN-4, PDQ) to define neuropathic pain is a conservative diagnostic criterion that may underestimate prevalence. According to the authors’ knowledge, this is the first study that has used three self-reported tools to determine neuropathic pain. Indeed, we found that the combination of all three questionnaires was more strongly correlated with VAS, as opposed to individual questionnaires, highlighting the importance of using different measures to detect pain type. Despite these, differences in psychological and behavioral outcomes between neuropathic and nociceptive patients with chronic LBP clearly showed worse scores in the neuropathic group, probably because of the underlying mechanisms of pain development [12]. As we stated in Section 1, nociceptive pain is often localized and proportional to mechanical triggers with no neurological features, while neuropathic pain is burning and characterized by dysesthesia, positive neurodynamic tests, and higher disability scores [12]. Thus, it is possible that nociceptive pain is clinically more bearable and easier to comprehend and treat. On the other hand, Tedeschi et al. [12] noted that ‘patients with neuropathic pain might require interventions beyond conventional mechanical therapies, such as targeted neurodynamic techniques or pharmacological approaches addressing nerve sensitization’. Nevertheless, the DN-4 and PDQ questionnaires represent a good starting point to detect a risky group of patients who may suffer from neuropathic pain. In that way, early detection and pain-specific management may be crucial for effective rehabilitation outcomes.

### 4.1. Study Limitations

Several limitations of this study should be acknowledged when interpreting the results. The study was conducted on a relatively limited sample of participants with chronic LMP, which may restrict the generalizability of the findings to the broader population of patients from different centers, races, and nationalities. Studies involving larger and more heterogeneous populations would provide more robust evidence regarding the prevalence of neuropathic pain and its associated factors. Neuropathic pain was identified using validated screening questionnaires (LANSS, DN4, and PDQ). Although these tools have demonstrated good sensitivity and specificity in previous studies [31,32,33,34,35,36,37,38,39,40,41], they are primarily screening instruments and do not represent definitive diagnostic methods. A comprehensive clinical evaluation combined with neurophysiological or imaging methods could provide a more precise classification of pain types. Nevertheless, we used validated versions of the LANNS [58], DN-4 [59] and PDQ [60]. Finally, variables such as depression, anxiety, QoL and sleep quality were assessed using self-reported questionnaires. Although these instruments are widely used in clinical research, they may be influenced by subjective perception and response bias. Despite these limitations, this study contributes to a better understanding of the prevalence of neuropathic pain in patients with chronic LBP and highlights its association with psychological and behavioral factors.

### 4.2. Perspective for Future Research

Future research should focus on longitudinal studies that would allow a better understanding of causal relationships between neuropathic pain, psychological symptoms, QoL and sleep quality in patients with chronic LBP. In addition, studies including larger and more diverse populations could provide more reliable estimates of the prevalence of neuropathic pain and identify potential demographic, clinical, and lifestyle factors associated with its development. Further research should also explore multidisciplinary intervention approaches that combine physical rehabilitation, psychological support, and sleep management strategies. Such approaches may contribute to improved functional outcomes and overall QoL in patients with neuropathic and nociceptive chronic LBP.

## 5. Conclusions

This study demonstrates that patients with a neuropathic component of pain exhibit poorer outcomes in mental health, QoL and sleep quality compared with patients with nociceptive pain when three independent validated questionnaires are used to differentiate between pain types. The findings suggest that the neuropathic component of pain significantly contributes to greater psychological burden and functional limitations in patients with chronic LBP. Therefore, healthcare professionals should consider these factors when monitoring patients with chronic LBP and when identifying potential lifestyle and psychosocial factors that may influence the severity of symptoms and overall functional status.

## Figures and Tables

**Table 1:** Basic descriptive statistics of the study participants, according to the type of pain; data are presented as mean ± SD or frequency (n) and percentage (%).

	Total (n = 150)	Nociceptive (n = 88)	Neuropathic (n = 62)	ES	*p*
Sex, n (%)	mean ± SD	mean ± SD	mean ± SD		
Men	83 (55.30%)	39 (44.30%)	44 (71.00%)		
Women	67 (44.70%)	49 (55.70%)	18 (29.00%)	0.26	**0.002**
Age (years)	46.55 ± 13.36	45.81 ± 13.38	47.60 ± 13.37	0.42	0.421
Height (cm)	178.06 ± 10.80	177.30 ± 9.98	179.15 ± 11.86	0.01	0.303
Weight (kg)	85.26 ± 15.53	82.74 ± 15.67	88.84 ± 14.73	0.04	**0.017**
BMI (kg/m^2^)	26.77 ± 3.63	26.13 ± 3.03	27.68 ± 4.21	0.04	**0.010**
IOTF cut points, n (%)					
Underweight	0 (0.00%)	0 (0.00%)	0 (0.00%)		
Normal weight	52 (34.70%)	37 (42.00%)	15 (24.20%)		
Overweight	77 (51.30%)	43 (48.90%)	34 (54.80%)		
Obesity	21 (14.00%)	8 (9.10%)	13 (21.00%)	0.22	**0.026**
Marital status, n (%)					
Single	30 (20.00%)	16 (18.20%)	14 (22.60%)		
Married	110 (73.30%)	64 (72.70%)	46 (74.20%)		
Divorced	2 (1.30%)	2 (2.30%)	0 (0.00%)		
Widowed	8 (5.30%)	6 (6.80%)	2 (3.20%)	0.13	0.448
Education, n (%)					
Primary school	3 (2.00%)	1 (1.10%)	2 (3.20%)		
Secondary school	80 (53.30%)	39 (44.30%)	41 (66.10%)		
Faculty (3 years)	26 (17.30%)	18 (20.50%)	8 (12.90%)		
Faculty (5 years)	30 (20.00%)	21 (23.90%)	9 (14.50%)		
Doctorate	11 (7.30%)	9 (10.20%)	2 (3.20%)	0.25	**0.048**
Occupation, n (%)					
Employed	108 (72.00%)	64 (72.70%)	44 (71.00%)		
Unemployed	24 (16.00%)	13 (14.80%)	11 (17.70%)		
Householder	8 (5.30%)	5 (5.70%)	3 (4.80%)		
Pensioner	10 (6.70%)	6 (6.80%)	4 (6.50%)	0.04	0.965
SES, n (%)					
Below average	31 (20.70%)	17 (19.30%)	14 (22.60%)		
Average	58 (38.70%)	34 (38.60%)	24 (38.70%)		
Above average	61 (47.60%)	37 (42.00%)	24 (38.70%)	0.04	0.866

ES: effect size (Hedges’s *G* and Kramer’s *V* for numerical and categorical data); BMI: body mass index; IOTF: International Obesity Task Force; SES: socioeconomic status; *p*: significance (<0.05).

**Table 2:** Pain-related differences in psychological outcomes of the study participants; data are presented as mean ± SD (median) or frequency (n) and percentage (%).

	Total (n = 150)	Nociceptive (n = 88)	Neuropathic (n = 62)	*p*
Psychological outcomes	Mean ± SD (median)	Mean ± SD (median)	Mean ± SD (median)	
HARS (score)	12.09 ± 8.13 (11.00)	10.30 ± 6.72 (10.00)	14.61 ± 9.28 (14.00)	**0.006**
HARS, n (%)				
Mild anxiety	91 (60.70%)	61 (69.30%)	30 (48.40%)	
Mild to moderate anxiety	32 (21.30%)	21 (23.90%)	11 (17.70%)	
Moderate to severe anxiety	19 (12.70%)	3 (3.40%)	16 (25.80%)	
Severe to very severe anxiety	8 (5.30%)	3 (3.40%)	5 (8.10%)	**<0.001**
BDI (score)	10.19 ± 6.93 (10.00)	8.70 ± 6.10 (9.00)	12.31 ± 7.52 (14.00)	**0.005**
BDI, n (%)				
Minimal depression	59 (39.30%)	41 (46.60%)	18 (29.00%)	
Mild depression	39 (26.00%)	25 (28.40%)	14 (22.60%)	
Moderate depression	48 (32.00%)	22 (25.00%)	26 (41.90%)	
Severe depression	4 (2.70%)	0 (0.00%)	4 (6.50%)	**0.004**

HARS: Hamilton Anxiety Rating Scale; BDI: Beck Depression Inventory; *p*: significance (<0.05).

**Table 3:** SF-36 domains according to the type of pain; data are presented as mean ± SD (median and interquartile range).

	Total (n = 150)	Nociceptive (n = 88)	Neuropathic (n = 62)	*p*
SF-36 domains	Mean ± SD (median)	Mean ± SD (median)	Mean ± SD (median)	
Physical functioning	60.50 ± 27.72 (65.00, 45.00–85.00)	65.68 ± 26.77 (65.00, 46.25–85.00)	53.15 ± 27.58 (50.00, 25.00–81.25)	**0.023**
Difficulty in role (physical)	49.50 ± 38.80 (50.00, 0.00–100.00)	58.81 ± 36.17 (50.00, 31.25–100.00)	36.29 ± 38.86 (25.00, 00.00–75.00)	**0.002**
Difficulty in role (emotional)	63.89 ± 37.76 (66.67, 33.33–100.00)	73.11 ± 34.60 (100.00, 66.67–100.00)	50.81 ± 38.46 (58.33, 0.00–100.00)	**0.003**
Vitality (energy)	52.77 ± 8.64 (55.00, 32.50–65.00)	53.86 ± 7.87 (55.00, 32.50–67.50)	51.21 ± 9.48 (50.00, 32.50–55.00)	**0.031**
Mental health	51.01 ± 10.40 (52.00, 48.00–56.00)	52.55 ± 10.21 (56.00, 48.00–60.00)	48.84 ± 10.37 (52.00, 48.00–53.00)	**0.043**
Social functioning	44.75 ± 20.49 (37.50, 45.00–60.00)	46.31 ± 18.20 (37.50, 37.50–62.50)	42.54 ± 22.46 (37.50, 25.00–62.50)	0.231
Pain	47.68 ± 21.53 (52.50, 48.00–56.00)	52.39 ± 22.58 (55.00, 50.00–60.00)	41.01 ± 18.09 (45.00, 45.00–55.00)	**<0.001**
General health	45.43 ± 8.21 (45.00, 40.00–50.00)	46.31 ± 8.11 (45.00, 40.00–50.00)	44.19 ± 8.26 (45.00, 40.00–50.00)	0.266
Global SF-36 score	51.94 ± 13.66 (51.68, 41.33–63.95)	56.13 ± 12.48 (57.20, 49.24–66.52)	46.00 ± 13.15 (46.80, 34.21–55.79)	**<0.001**

SF-36: 36-Item Short Form Survey; *p*: significance (<0.05).

**Table 4:** Sleep quality domains according to the type of pain; data are presented as mean ± SD (median and interquartile range) or frequency (n) and percentage (%).

	Total (n = 150)	Nociceptive (n = 88)	Neuropathic (n = 62)	*p*
PSQI domains	mean ± SD (median)	mean ± SD (median)	mean ± SD (median)	
Sleep latency	1.47 ± 1.01 (2.00, 0.00–2.00)	1.49 ± 1.04 (2.00, 0.00–2.00)	1.44 ± 0.99 (2.00, 0.00–2.00)	0.591
Sleep duration	0.73 ± 0.90 (0.00, 0.00–1.00)	0.56 ± 0.88 (0.00, 0.00–1.00)	0.98 ± 0.88 (1.00, 0.00–2.00)	**0.010**
Sleep efficiency	0.83 ± 1.11 (0.00, 0.00–2.00)	0.61 ± 0.98 (0.00, 0.00–1.00)	1.15 ± 1.23 (1.00, 0.00–2.25)	**0.006**
Sleep disturbances	1.30 ± 0.63 (1.00, 1.00–2.00)	1.13 ± 0.52 (1.00, 1.00–1.00)	1.55 ± 0.69 (1.50, 1.00–2.00)	**<0.001**
Sleep medication	0.29 ± 0.61 (0.00, 0.00–0.00)	0.16 ± 0.40 (0.00, 0.00–0.00)	0.48 ± 0.78 (0.00, 0.00–1.00)	**0.003**
Sleep quality	1.05 ± 0.92 (1.00, 0.00–2.00)	0.78 ± 0.81 (1.00, 0.00–1.00)	1.44 ± 0.93 1.00, 1.00–2.00)	**<0.001**
Sleep dysfunction	0.80 ± 0.65 (1.00, 0.00–1.00)	0.60 ± 0.58 (1.00, 0.00–1.00)	1.08 ± 0.64 (1.00, 0.00–1.25)	**<0.001**
Global PSQI score	6.48 ± 3.89 (6.00, 4.00–9.00)	5.33 ± 3.55 (5.00, 3.25–6.00)	8.11 ± 3.77 (8.00, 6.00–11.00)	**0.001**
Sleep quality				
Good (≤5 points)	71 (47.30%)	58 (65.90%)	13 (21.00%)	
Poor (>5 points)	79 (52.70%)	30 (34.10%)	49 (79.00%)	**0.001**

PSQI: Pittsburgh Sleep Quality Questionnaire; *p*: significance (<0.05).

## Data Availability

The datasets used and/or analyzed during the current study are available from the author upon reasonable request.
